# The Amination of Waste Newsprint Paper with Various Aminating Agents (Ammonia Water, Ethylenediamine, and Diethylenetriamine) to Improve the Sorption Efficiency of Anionic Dyes

**DOI:** 10.3390/molecules29246024

**Published:** 2024-12-20

**Authors:** Tomasz Jóźwiak

**Affiliations:** Department of Environmental Engineering, University of Warmia and Mazury in Olsztyn, Warszawska St. 117a, 10-957 Olsztyn, Poland; tomasz.jozwiak@uwm.edu.pl

**Keywords:** waste paper, newsprint, unconventional sorbent, amination, ammonia, ethylenediamine, diethylenetriamine, sorption, anionic dyes, reactive dyes

## Abstract

This study aimed to investigate the effect of aminating waste newsprint paper with different aminating agents (ammonia/ammonia water, ethylenediamine, and diethylenetriamine) for the sorption efficiency of Reactive Black 5 (RB5) and Reactive Yellow 84 (RY84) dyes. To increase the amination efficiency, the paper material was pre-activated with epichlorohydrin. The scope of this study included the characterization of the sorbents tested (FTIR, elemental analysis, BET surface area, porosity, and pH_PZC_), determination of the influence of pH on dye sorption efficiency, sorption kinetics, and the maximum sorption capacity of the dyes. The study results showed that amination with ethylenediamine and diethylenetriamine introduced 87% and 194% more amine groups into the sorbent’s structure compared to the treatment with ammonia. The sorption efficiency of RB5 and RY84 on the sorbents tested was the highest in the pH range of 2–3. The sorption equilibrium time ranged from 90 to 150 min and was longer in the case of the unmodified sorbents. The experimental data from the study were best described by the pseudo-second-order model and the Langmuir 1 and 2 models. Amination had a very strong effect on the sorption capacity of newsprint. For example, the sorption capacity of newsprint paper towards RB5 (Q_max_ = 7.12 mg/g) increased after amination with ammonia, ethylenediamine, and diethylenetriamine to the value of Q_max_ = 182.78 mg/g, Q_max_ = 202.7 mg/g, and Q_max_ = 231.5 mg/g, respectively.

## 1. Introduction

Dyes can selectively absorb electromagnetic radiation in the visible light range (from 380 to 780 nm), which imparts a color effect to them. They are often used in industry to color everyday objects. Their annual global production reaches about 1.3 million tons [1] and extends to approximately 10,000 different dye types [2,3] that are extensively used in the textile, tanning, and paper industries [4,5].

Due to the imperfections of the dyeing methods currently in use, a significant proportion of the dyes used in this process do not bind with the material and end up in post-production wastewater. This is particularly true for anionic reactive dyes, whose losses during dyeing range from 10% to even 50% [6]. Incorrectly managed colored industrial wastewater can pose a significant threat to the natural environment. The dyes used in industry are generally organic compounds with a complex chemical structure, which makes them resistant to biodegradation [7]. For this reason, their significant proportion can persist in the environment for a long time [8]. Pollution with dyes is particularly dangerous for natural waters. Dyes dissolved in water restrict the access of aquatic autotrophs to sunlight, resulting in a low efficiency of primary production [9]. Some dyes can react with dissolved oxygen in the water, which—in combination with inhibited photosynthesis—can lead to the development of anaerobic conditions in the reservoir [10]. Individual dyes can also be toxic to aquatic flora and fauna [11]. Consequently, environmental contamination with dyes can lead to the collapse of the local aquatic ecosystem.

The prospect of environmental degradation upon contamination with dyes should prompt the use of effective technologies for the treatment of colored wastewater. Sorption is generally believed to be one of the most effective and environmentally friendly methods for wastewater decolorization [12,13,14], which consists of binding pollutants (sorbates) to a sorption material (sorbent) [15]. The prerequisite for high process efficiency is the use of a high-quality sorbent and the selection of suitable process parameters (pH value, time, and temperature) [16]. 

Popular sorbents are those based on activated carbons, which have an exceptionally large specific surface area, often >1000 m^2^/g [17,18], ensuring their good sorption properties towards most types of dyes. Another advantage is the possibility of their regeneration and reuse [19], while their drawbacks include a high market price and considerable costs of regeneration [20].

Cheaper substitutes for activated carbon are currently being sought. In principle, the raw material for the production of an alternative sorbent should not only be inexpensive but also widely available. Great hopes are therefore being pinned on the production of sorbents from waste materials generated in the agricultural and food industries [21]. In recent years, many materials derived from plant waste biomass have been tested for their sorption properties, namely crop leaves [22,23,24], seed husks [25,26,27,28,29], nut shells [30,31,32], and vegetable and fruit peels [33,34,35,36]. Many studies have also looked at the feasibility of removing dyes from waste materials from the wood industry, such as sawdust [37,38,39], bark [40,41], cones [42,43], or tree leaves [44,45,46]. Scientists agree that the sorption properties of plant biomass are mainly related to the content of its polysaccharides (cellulose and hemicellulose) and lignin. 

A disadvantage of sorbents made from plant waste is their likely contents of easily biodegradable saccharides (starch, pectins, sucrose, and fructose), proteins, and plant pigments (chlorophylls and carotenoids), which pose the risk of secondary contamination of wastewater. In addition, these materials are often characterized by a short shelf life and are susceptible to biodegradation, which curbs their applicability.

Another waste product based on cellulose with a possible admixture of lignin is waste paper. This includes surplus or used paper products that can serve as a raw material for paper production [47]. Waste paper materials have no readily biodegradable components and are more durable than plant biomass. The amount of waste paper collected annually worldwide reaches 250 million tons (2022) [48]. Its collection for recycling is common in many countries around the world, which is why it can not only be considered cheap but also widely available.

The efficiency of dye sorption on waste paper depends mainly on the paper type and other factors, like lignin content, basis weight, and the chemical composition of fillers and coatings. The results of studies comparing the sorption properties of different types of recovered paper (newsprint, coated/glossy paper, office paper, and corrugated board) showed the highest efficiency of dye sorption by newsprint [49]. The authors of the above-mentioned article showed that a relatively high lignin content (up to 30%) and a relatively low basis weight (50 g/m^2^) positively affected the sorption properties of newsprint, which was reflected in easier access to the sorption centers. The attractiveness of newsprint as a sorption material is increased by the fact that it is one of the cheapest types of paper. It is mainly made from softwood pulp, which is not delignified for economic reasons. The annual production of this material is 22 million tons (2022) [50]. Although used newsprint contains certain amounts of printing ink, the risk of its release and secondary contamination of aqueous solutions is very low. This is confirmed by a study conducted in 2024 on the effectiveness of dyeing aqueous solutions with different types of waste paper [49].

A disadvantage of newsprint and other lignocellulosic materials is the relatively low efficiency of sorption of anionic dyes (acidic and reactive). This hinders the possibility of using these sorbents for the treatment of real-colored wastewater, as anionic dyes are the most common type of contaminant. The reason for the low efficiency of sorption of anionic dyes on lignocellulosic materials is usually their neutral or acidic nature, which instead facilitates the sorption of basic (cationic) dyes [51].

The efficiency of the sorption of anionic dyes on lignocellulosic materials can be increased by their chemical modification, which consists of introducing basic functional groups into the sorbent’s structure [52,53]. These groups are important sorption centers for most sorbates that have a negative charge in aqueous solutions. One way of enriching the sorbent with basic functional groups is via amination, which consists of reacting the material with an aminating agent, thanks to which the sorbent gains amino groups. The simplest aminating agent is ammonia in the form of ammonia water (25–30%). Modification with ammonia leads to the acquisition of primary functional groups by the sorbent’s structure [54]. The direct reaction of lignocellulosic material with ammonia water is not very efficient under normal conditions (25 °C, 100 kPa). However, the efficiency of such amination can be significantly increased by pre-activating the material with epichlorohydrin [55]. So far, studies have been conducted on the use of ammonia water for the amination of materials such as cotton fibers [52], sunflower husks [26], buckwheat [53], rapeseed hulls [55], wheat straw [56], and goldenrod biomass [54]. The efficiency of dye sorption on the above-mentioned sorbents has been substantially boosted after modification.

**Figure 1:**
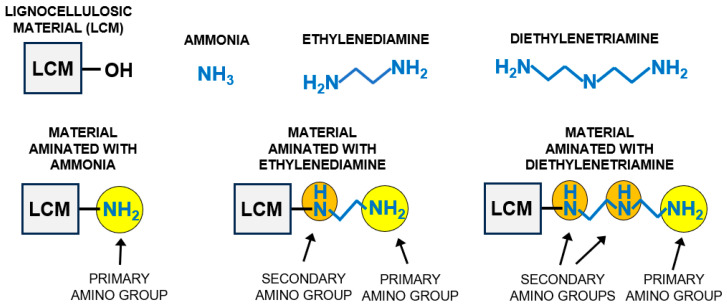
Structural formulas of aminating agents (ammonia, ethylenediamine, and diethylenetriamine) and a simplified diagram of materials modified with selected aminating agents.

Theoretically, any substance containing amine groups can be used as an aminating agent. Alternative compounds that can serve this function include, e.g., ethylenediamine, which has two amine groups, and diethylenetriamine, which has three amine groups (Figure 1). Theoretically, the amination of the material with ethylenediamine and diethylenetriamine could lead to two and three times more amine groups being introduced into the structure of the sorbent compared to the ammonia treatment (Figure 1). A larger number of functional amine groups on the sorbent’s surface could lead to a higher sorption capacity for anionic dyes. However, it should be kept in mind that if the material is aminated with ethylenediamine or diethylenetriamine, a significant proportion of the added amine groups would be secondary. Although secondary amino groups are ionizable and can serve as an active site for anionic dyes, they are likely less accessible than the primary amino groups. To the best of the authors’ knowledge, no research results on the efficiency of sorbents modified with various amination agents have been published so far.

The study described in this article investigated the effect of three different aminating agents (ammonia water, ethylenediamine, and diethylenetriamine) on the sorption properties of waste newsprint paper towards common industrial reactive dyes (Reactive Black 5 and Reactive Yellow 84).

## 2. Results and Discussion

### 2.1. Characterization of the Tested Sorbents (FTIR Analysis, Specific Surface Area, and Elementary Analysis)

The following sorbents were tested in this work:

- Unmodified newsprint paper (N);
- Newsprint paper pre-activated with epichlorohydrin (NE);
- Newsprint paper pre-activated with epichlorohydrin and next aminated with ammonia water (NEN1);
- Newsprint paper pre-activated with epichlorohydrin and next aminated with ethylenediamine (NEN2);
- Newsprint paper pre-activated with epichlorohydrin and next aminated with diethylenetriamine (NEN3).

#### 2.1.1. FTIR Analysis

**Figure 2:**
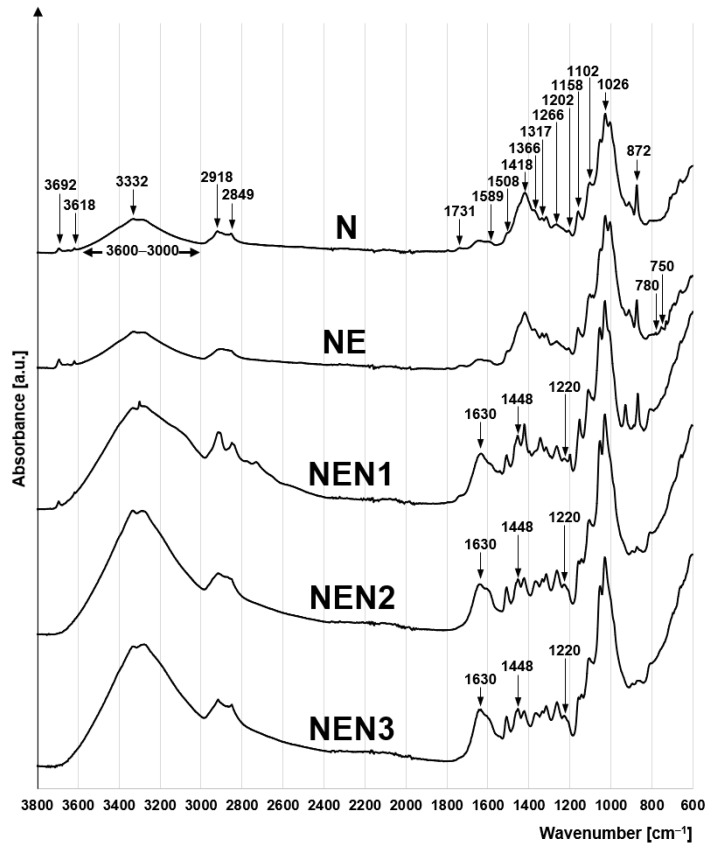
FTIR spectra of N, NE, NEN1, NEN2, and NEN3.

The FTIR spectra of the sorbents tested are typical of lignocellulosic materials (Figure 2). The peaks at 1202 cm^−1^, 1158 cm^−1^, 1102 cm^−1,^ and 1026 cm^−1^ indicate the presence of a C–O–C glycosidic bond of the saccharide rings of cellulose and hemicellulose [57,58]. The peak at 1052 cm^−1^ corresponds to the stretching of the C-OH bond of the secondary alcohol groups of cellulose [59]. Also, characteristic of holocelluloses include the peaks at 1366 cm^−1^ and 1317 cm^−1^, which correspond to the rocking and bending vibrations of -CH_2_ on the C6 carbon of the saccharide ring [60], and a distinct peak at 1418 cm^−1^, which indicates the stretching vibrations of –CH_2_ [61].

The presence of lignin in the material is evidenced by the peaks at 1508 cm^−1^ and 1261 cm^−1^, which correspond to the C=C bonds of the phenylpropane rings typical of lignin [62] (Figure 2). The lignin content in the analyzed materials is also associated with the peaks at 1731 cm^−1^ and 1589 cm^−1^, which indicate the presence of carbonyl C=O bonds [63]. The peaks at 2918 cm^−1^ and 2849 cm^−1^ can be ascribed to the asymmetric and symmetric stretching vibrations of the –CH_2_ groups of the aliphatic side chains of lignin [63]. In turn, a broad band at 3500–3000 cm^−1^ corresponds to the stretching of the O–H bond of hydroxyl functional groups present in both cellulose and lignin, whereas a small peak visible at 3332 cm^−1^ can be ascribed to the stretching of the N-H bond (amide A) [64]. The presence of this bond is due to the presence of small amounts of proteins in the material that were not removed from the pulp during the production of newsprint paper.

The peak at 872 cm^−1^ visible in each spectrum is ascribed to the asymmetric bending vibration of the CO bond of carbonates [65] (Figure 2), which is due to the presence of calcite (CaCO_3_) in the material, which is commonly added as a filler to paper products. In addition to calcite, newsprint also contains some amounts of kaolin. This is evidenced by the peaks that are characteristic for this aluminosilicate visible at 3692 cm^−1^ and 3618 cm^−1^, which correspond to the O–H and Al–OH bonds [66].

The NE spectrum reveals small peaks at 780 cm^−1^ and 750 cm^−1^, indicating the presence of epoxy groups in the sorbent [67,68], which is due to the material reaction with epichlorohydrin.

Characteristics of the NEN1, NEN2, and NEN3 spectra are the peaks at 1448 cm^−1^ and 1220 cm^−1^, indicating the stretching of the C–N bond, and the peak at 1630 cm^−1,^ which can be ascribed to the shear vibrations of NH_2_ [69,70] (Figure 2). The presence of these peaks confirms that the modified material has undergone the amination process.

#### 2.1.2. Elemental Analysis

The elemental analysis of the sorbents tested showed that the percentage of nitrogen in the material increased in the following order: N < NEN1 < NEN2 < NEN3 (Table 1). When newsprint was aminated with ethylenediamine, almost twice as much nitrogen was introduced into the material as when the paper was modified with ammonia (ammonia water) (87% more). When diethylenetriamine was used, the amount of nitrogen introduced was almost three times as high (194% more) as ammonia water treatment. The results obtained are consistent with the predicted numbers of amine groups introduced, which are shown in Figure 1.

#### 2.1.3. Analysis of Specific Surface Area and Porosity

The surface and porosity parameters of the sorbents analyzed in this study are listed in Table 2. Newsprint (N) is a material with a very small specific surface area of about 0.5 m^2^/g and a small pore volume of ~0.002 cm^3^/g (Table 2). Other types of waste paper have much larger surface areas, e.g., office paper (BET surface area 1.3 m^2^/g, pore volume ~0.01 cm^3^/g) or high gloss paper (LCW) from high gloss magazines (BET surface area 2.4 m^2^/g, pore volume ~0.02 cm^3^/g) [49]. This is probably due to the special method employed in newsprint production, in which the material is heavily pressed.

Modifications of N entailing its treatment with epichlorohydrin or aminating agents had no significant effect on its surface area or porosity (Table 2). There were also no clear correlations between the surface area of the modified material and its mass or type of functional groups. This is due to the fact that chemical modifications interfered with the material only at the molecular level. The enrichment of the sorbent with additional functional groups had no major influence on its spatial structure. For this reason, it is not necessary to analyze the SEM images of the modified paper-based sorbents.

A small specific surface area of the sorbent does not necessarily mean its poor sorption performance. As previous studies have shown, comparing the sorption capacity of different grades of recovered paper, newsprint showed the highest sorption capacity for dyes (compared to office paper, LCW paper, and corrugated board) despite its smallest specific surface area [49]. Theoretically, the structure of sorbents based on recovered paper can loosen after being introduced into water, which can lead to increased availability of sorption centers for dyes. However, the sorption of dyes is probably more strongly affected by the chemical composition of the waste paper, in particular by the content of functional groups with which dyes can interact.

### 2.2. Effect of pH on the Efficiency of Dye Sorption

**Figure 3:**
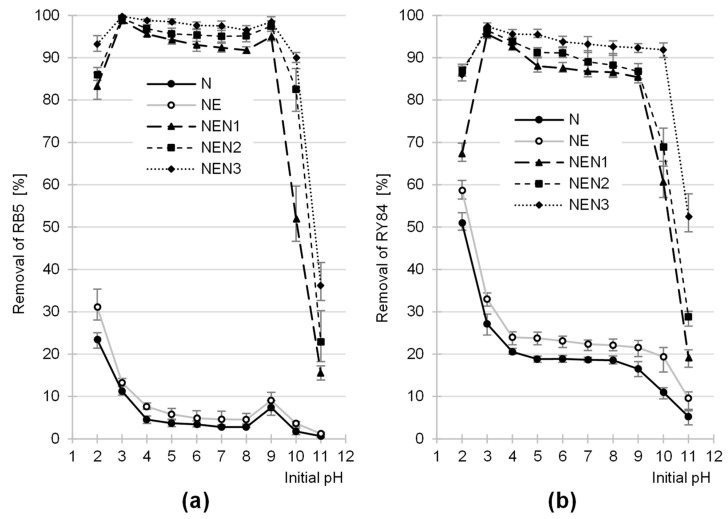
Effect of pH on the sorption efficiency of (**a**) RB5 and (**b**) RY84 onto tested sorbents (average + range). Temp. 25 °C.

The sorption efficiency of RB5 and RY84 on N and NE was the highest at pH 2, whereas their sorption on the aminated sorbents (NEN1, NEN2, and NEN3) was the highest at pH 3 (Figure 3). In general, the sorption efficiency of the tested dyes decreased with increasing pH. For N and NE, the greatest decrease in binding efficiency of RB5 and RY84 was observed with pH increasing from pH 2 to pH 4, while in the initial pH range of 4–10, the sorption efficiency of the dyes remained at a similar, low level. Sorbents based on aminated paper (NEN1, NEN2, and NEN3) exhibited a high sorption efficiency in a broad pH range (pH 2–9) in contrast to the non-aminated sorbents (N and NE) (Figure 3).

In the analytical series with RB5, a slight increase was observed in the sorption efficiency at pH 9. For each sorbent tested, the sorption efficiency of RB5 and RY84 was the lowest at pH 11. Regardless of the initial pH, the sorption efficiency of the dyes on the tested sorbents increased in the following order: N < NE < NEN1 < NEN2 < NEN3.

The high sorption efficiency of the anionic dyes at low pH resulted from the acquisition of a positive charge by the sorbent’s surface. With an excess of hydronium ions in the solution, some functional groups were protonated. In the case of N and NE, these were mainly hydroxyl groups (–OH) derived from polysaccharides and lignins. In the case of NEN1, NEN2, and NEN3, the amine groups (primary –NH_2_ and secondary –NH–) added during amination could also be ionized in addition to the hydroxyl groups.

–OH + H_3_O^+^ →–OH_2_^+^ + H_2_O

–NH_2_ + H_3_O^+^ →–NH_3_^+^ + H_2_O

–NH^−^ + H_3_O^+^ →–NH_2_^+^– + H_2_O

Positively charged functional groups present on the surface of the sorbents attracted anionic dyes electrostatically, which enhanced their sorption.

In general, the hydroxyl groups of lignocellulosic materials are hardly susceptible to ionization. Their protonation is possible at a very low pH (pH 2–3), while most -OH groups remain in the unprotonated form in the pH range of 4–10. This explains the significant decrease in the sorption capacity of RB5 and RY84 on N and NE observed in the pH range of 2–4 (Figure 3). Amine groups, on the other hand, undergo protonation even in a slightly alkaline environment (up to pH 9.5). Sorbents enriched with amine functional groups (NEN1, NEN2, and NEN3) exhibited a large number of local positive charges at pH 2–9, which explains the high sorption efficiency of the anionic dyes in this broad pH range. The lower sorption efficiency of RB5 and RY84 determined at pH 2 than at pH 3 for the aminated sorbents was probably due to the fact that at pH 2, some of the sulfone groups present in the structure of the dyes were deionized. This led to weaker electrostatic interactions of RB5/RY84 with the surface of the sorbents and, thus, to a lower efficiency of their sorption.

–SO_3_^−^ + H_3_O^+^ →–SO_3_H + H_2_O

The efficiency of protonation of the functional groups of the sorbent was observed to decrease with increasing pH. The deprotonation of some functional groups can occur under alkaline conditions with a high concentration of hydroxyl ions. At pH 11, both hydroxyl and amine groups could occur in the deprotonated form. Negatively charged functional groups present on the sorbent’s surface repelled the anions of the dyes electrostatically, which explains the strong inhibition of their sorption (Figure 3).

–OH + OH^−^ → –O^−^ + H_2_O

–NH_2_ + OH^−^ → –NH^−^ + H_2_O

A high sorption efficiency of anionic dyes at low pH was also observed in the studies on the sorption of Reactive Black 5 on such sorbents as goldenrod biomass [54], buckwheat hulls [53] and activated carbon [71]. In the case of Reactive Yellow 84, a positive effect of low pH was also demonstrated in the studies on its sorption on cotton fibers [52], sunflower seed hulls [26], and compost [72].

Some increase in the sorption efficiency of RB5 at pH 9 (Figure 3a) was probably caused by the presence of an amine group in the structure of this dye. At pH 9, the surface of the sorbents was probably already negatively charged (the total number of negative charges was higher than that of the positive charges) (which will be confirmed by the results of studies on the pH_PZC_ of the sorbents presented later in the article). On the other hand, at this pH, a significant portion of the RB5 molecules had protonated amine groups. The interaction between the negatively charged surface and the –NH_3_^+^ group of the dye could theoretically increase its sorption performance. The result obtained, manifested in the increase in RB5 sorption performance at pH 9, is typical of the sorbents with numerous amine groups, e.g., chitin [73] or chitosan sorbents [74,75], or aminated plant biomass [54,56]. 

**Figure 4:**
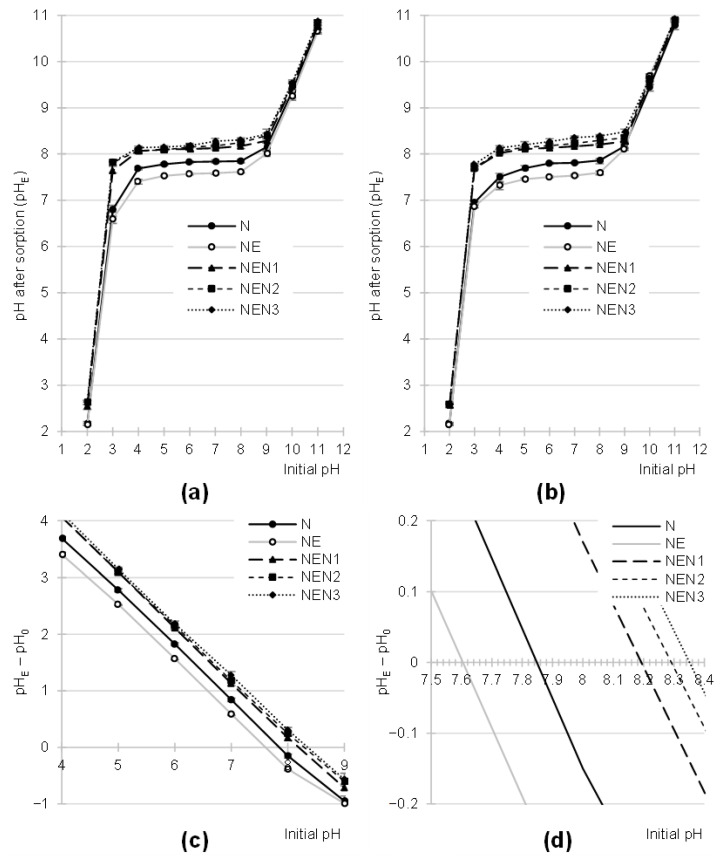
Changes in pH values of the solutions during sorption of (**a**) RB5 and (**b**) RY84 onto the tested sorbents (average + range). (**c**,**d**) pH_PZC_ of the tested sorbents determined with the “drift” method. Temp. 25 °C.

The paper-based sorbents analyzed in this study affected the change in the pH of dye solutions. For example, with an initial pH range of 3–9, the pH after 2 h of sorption ranged from 6.80 to 8.15 for N, from 6.60 to 8.01 for NE, from 7.64 to 8.29 for NEN1, from 7.82 to 8.40 for NEN2, and from 7.83 to 8.43 for NEN3 (Figure 4a,b).

The changes in the pH values of the solutions observed in the systems are due to the fact that the sorbents contain functional groups capable of ionization. As mentioned above, some functional groups were protonated at low pH. One of the effects of this process was a decrease in the concentration of hydronium ions and thus an increase in the pH value of the solution. Conversely, at a high pH value, some functional groups were deprotonated, which led to a decrease in the concentration of hydroxyl ions and thus to a decrease in the pH value of the solution. The final pH value in the system always depends on the ratio between the alkaline and acidic groups on the surface of the sorbent. The system always tends to a solution pH that is close to the pH_PZC_ of the sorbent (PZC—point of zero charge). It can be said that the pH_PZC_ is the pH value at which the surface of the sorbent has a neutral charge (the number of protonated groups is equal to the number of deprotonated groups). The pH_PZC_ values determined by the “drift” method in the present study were pH_PZC_ = 7.84 for N, pH_PZC_ = 7.60 for NE, pH_PZC_ = 8.20 for NEN1, pH_PZC_ = 8.30 for NEN2, and pH_PZC_ = 8.34 for NEN3 (Figure 4c,d).

The pH_PZC_ value of the sorbents increased in the following order: N < NEN1 < NEN2 < NEN3. This order is identical to the order of nitrogen contents in the material (described in Section 2.1) and results from the increasing number of amine functional groups in this series, which have a basic nature.

The sorption efficiency of anionic dyes on the analyzed sorbents was the highest at a pH of 2–3. Since colored wastewater usually has pH > 2, further analyses (described in Section 2.3 and Section 2.4) were carried out at pH 3.

### 2.3. Kinetics of RB5 and RY84 Sorption onto Tested Sorbents

**Figure 5:**
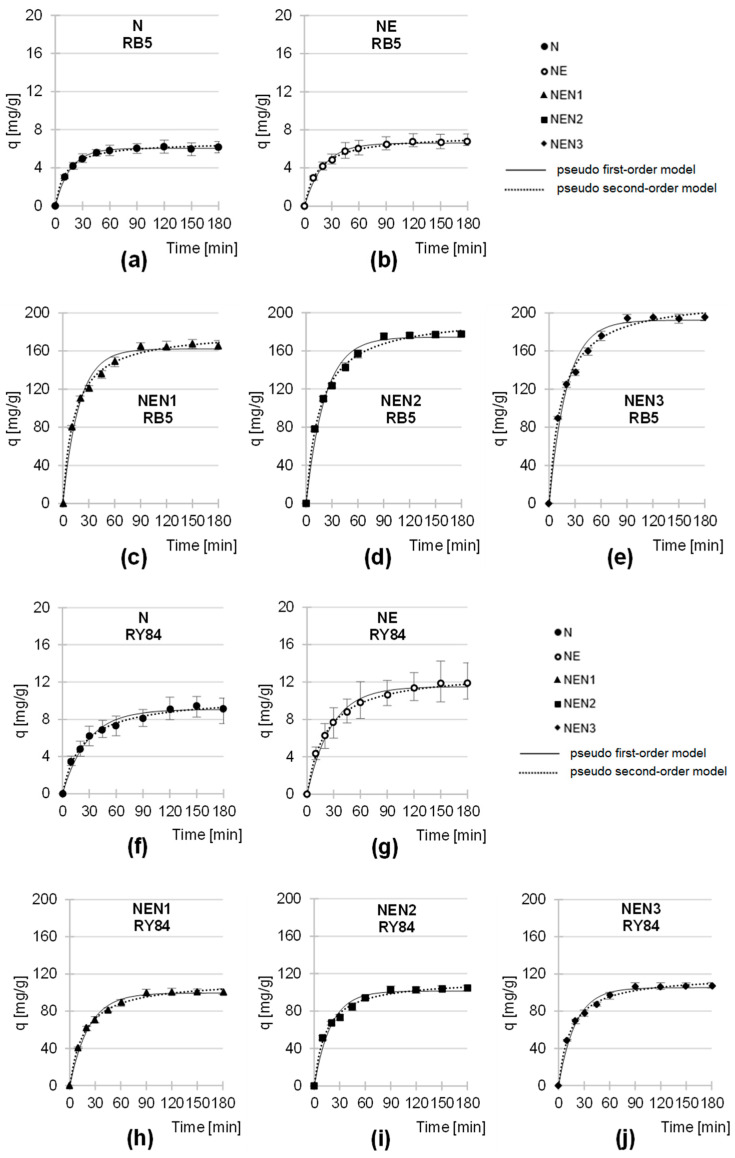
Sorption kinetics of RB5 sorption onto (**a**) N, (**b**) NE, (**c**) NEN1, (**d**) NEN2, and (**e**) NEN3; and of RY84 sorption onto (**f**) N, (**g**) NE, (**h**) NEN1, (**i**) NEN2, and (**j**) NEN3, (average). Pseudo-first-order model and pseudo-second-order model. Temp. 25 °C.

The equilibrium time of the sorption of anionic dyes depended on the type of sorbent. For N and NE, the sorption of RB5 and RY84 was “completed” after 120–150 min, while for the aminated sorbents (NEN1, NEN2, and NEN3), the system reached equilibrium after 90 min (Figure 5, Table 3). The efficiency of dye binding on the analyzed sorbents was the highest at the beginning of the process. After the first 20 min of sorption, the amount of RB5 dye bound to the sorbents was between 61.8 and 67.4% of the q_e_ value (q_e_—the amount of dye sorbed after the equilibration time), and, in the case of RY84, it was between 51.0 and 64.9% q_e_.

Sorption equilibrium times of 120–150 min were also reported in the studies investigating RB5 removal on activated carbon from carob tree (120 min) [76] and goldenrod biomass (150 min) [54]. On the other hand, significantly shorter sorption equilibrium times (similar to those recorded on aminated sorbents) were obtained in experiments with RB5 sorption on activated carbon from bamboo (60 min) [77] and biochar from gasification residues (90 min) [78], and also during sorption of RY84 on sunflower seed hulls (90 min) [26].

The slightly longer sorption times of RY84 compared to RB5 observed in the research series with N and NE could be due to the higher molecular weight of RY84. The larger dimensions of the RY84 molecules could slow down the process of their binding to the sorption centers in deeper layers of the sorbent.

The generally shorter equilibrium times of dye sorption on NEN1, NEN2, and NEN3 compared to N and NE are probably due to a larger number of protonated functional groups on the surface of the aminated sorbents and, thus, to a larger overall positive charge. A stronger charge on the surface of the sorbent materials could accelerate the sorption of anionic dyes and consequently shorten the time in which the system reached the equilibrium state.

The experimental data from the studies on the kinetics of dye sorption were described with pseudo-first and pseudo-second-order models (Figure 5, Table 3). In each series of the study, the pseudo-first-order model showed the greatest fit to experimental data, regardless of the type of sorbent tested. This is a typical result for the sorption of organic dyes onto lignocellulosic sorbents and was also observed in the studies on the kinetics of RB5 sorption onto goldenrod biomass [54], sunflower seed hulls [26], buckwheat hulls [53], or wheat straw [56].

**Figure 6:**
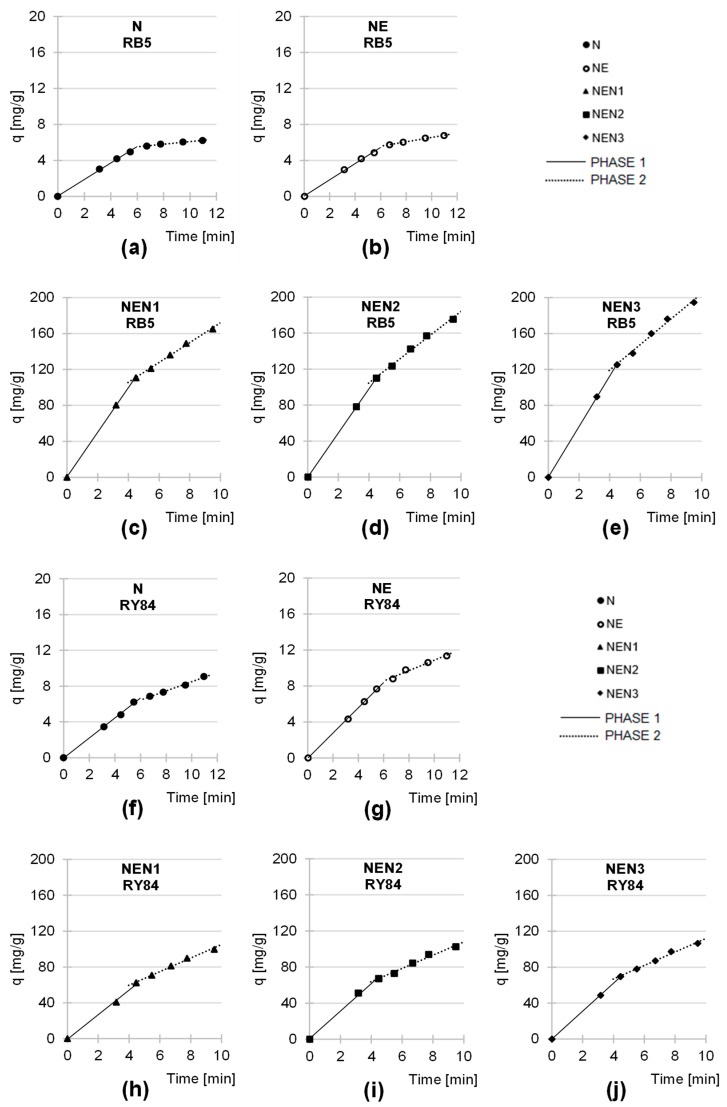
Intraparticle diffusion model of RB5 sorption onto (**a**) N, (**b**) NE, (**c**) NEN1, (**d**) NEN2, and (**e**) NEN3; and of RY84 sorption onto (**f**) N, (**g**) NE, (**h**) NEN1, (**i**) NEN2, and (**j**) NEN3, (average). Temp. 25 °C.

The data from the sorption kinetics studies were also described using the intraparticle diffusion model (Figure 6, Table 4). The results of the analyses of this model show that the sorption of RB5 and RY84 on the sorbents based on newsprint paper occurred in two main phases, differing in intensity and duration.

Presumably, in the first phase of sorption, which is characterized by high intensity and short duration, anions of dyes diffused from the solution onto the surface of the sorbent and bound to the most accessible active sites of the sorption material. Once most of the active sites were saturated on the sorbent’s surface, the second phase began. In this phase, the last free sites available on the surface of the sorbent material were filled, and the dye anions also attached themselves to the sorption centers in the deeper layers of the sorbent. Phase 2 was characterized by strong competition between the dye anions for the last unoccupied active sites. In addition, the penetration of the dyes into the less accessible sorption centers was quite time-consuming. For this reason, phase 2 was generally characterized by a lower sorption efficiency than phase 1 and was also longer.

The shorter duration of the first major sorption phase in the research series with NEN1, NEN2, and NEN3 compared to N and NE is probably due to the higher positive charge on the surface of the aminated sorbents, as mentioned earlier in this paper.

The values of q_e_ and k_2_ determined from the pseudo-second-order model (Table 3) and the values of k_d1_ and k_d2_ determined from the intraparticle diffusion model (Table 4) indicate that both the rate and efficiency of sorption of the anionic dyes increased in the order: N < NE < NEN1 < NEN2 < NEN3. The higher intensity of RB5 and RY84 sorption on NE compared to N probably resulted from the epoxide functional groups bound to the sorbent’s structure, which can interact with the dyes. Also, the chemisorption of dyes on NE cannot be excluded due to the relatively high reactivity of epoxy groups. However, the increase in the efficiency of dye sorption on the sorbent after its activation with epichlorohydrin was relatively low. Significantly better results were obtained for the sorbents that were first modified with epichlorohydrin and then subjected to the amination process (NEN1, NEN2, and NEN3). For each aminating agent used, the efficiency of the sorbents increased at least 10-fold (Table 3 and Table 4). As a result of amination, many amine groups were added to the sorbent’s structure, which represents important sorption centers for anionic dyes. In turn, the number of amine groups on N and NE was very low and mainly related to the presence of traces of proteins in the mechanical pulp (raw material for newsprint production). This explains the observed difference in the sorption performance between the aminated (NEN1, NEN2, and NEN3) and the non-aminated (N and NE) sorbents.

The amination efficiency of the sorbents depended on the aminating agent type. The elemental analysis confirmed that the amount of nitrogen added (in the form of amine groups) increased in the following order: NEN1 < NEN2 < NEN3. As expected, as the amount of amine functional groups increased in the material, its ability to sorb anionic dyes also increased, which explains the observed range of efficiencies of the aminated sorbents (NEN1 < NEN2 < NEN3).

It was also found that there were no clear correlations between the specific surface area and porosity of the sorbents tested (Table 2) and the kinetics of dye sorption on the sorbents.

### 2.4. Maximum Sorption Capacity of the Tested Sorbents

**Figure 7:**
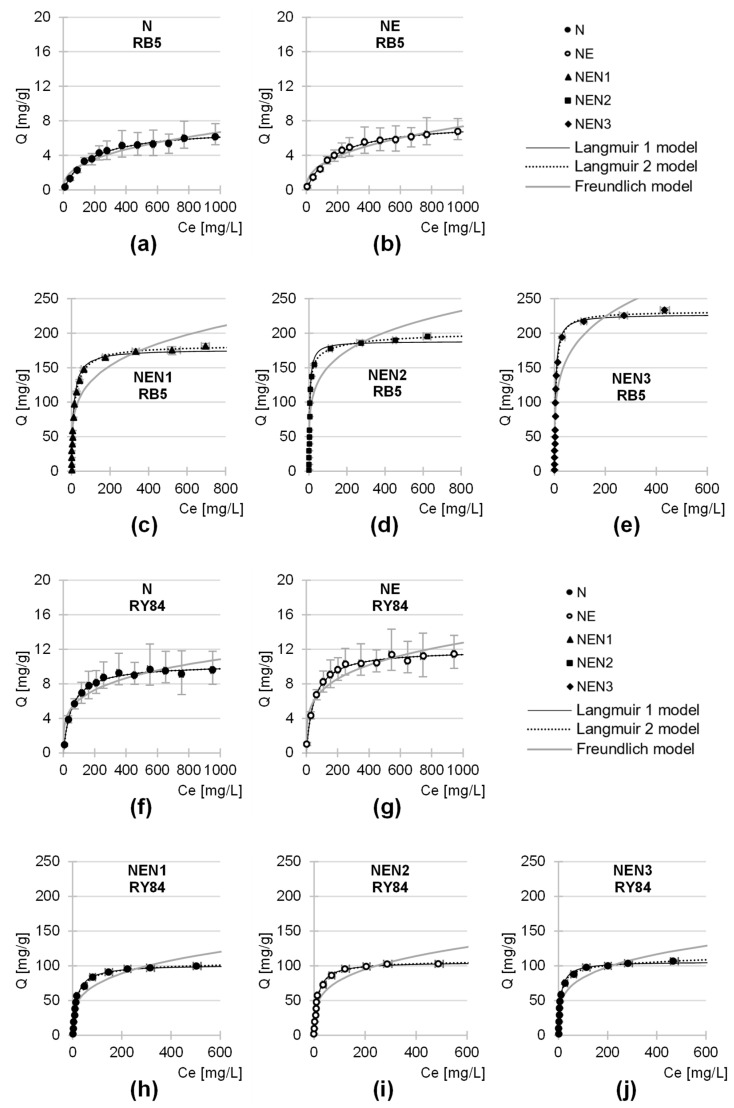
Isotherm of RB5 sorption onto: (**a**) N, (**b**) NE, (**c**) NEN1, (**d**) NEN2, and (**e**) NEN3; and RY84 sorption onto: (**f**) N, (**g**) NE, (**h**) NEN1, (**i**) NEN2, and (**j**) NEN3, (average + range). Langmuir 1, Langmuir 2, and Freundlich models. Temp. 25 °C.

Experimental data from studies on the maximum sorption capacity of the sorbents tested were described with three popular sorption models: Langmuir 1, Langmuir 2, and Freundlich isotherms (Figure 7; Table 5). In all analytical series, the Langmuir 1 and 2 models showed a better fit to the experimental data than the Freundlich model. This finding indicates that the sorption mechanism of the dye is better explained by the Langmuir theory, according to which only one sorbate molecule can bind to an active site. Therefore, the dye anions form the so-called “monolayer” on the sorbent’s surface. The bond between the sorbate and the active site of the sorbent is not permanent. Therefore, these dyes can migrate across this monolayer, i.e., from the surface to active sites that are located, e.g., in deeper layers of the sorbent (e.g., in pores). Dye exchange between the monolayer and the solution is also likely (which can occur especially after sorption equilibrium has been reached).

In the analytical series with N and NE, the values of Q_max_, K_C_/K_1_/K_2,_ and R_2_ determined from the Langmuir 1 and Langmuir 2 models were the same (Table 5). This finding indicates that only one type of active site played an important role in RB5 and RY84 sorption on these sorbents. Presumably, these were the protonated hydroxyl groups that interacted electrostatically with the dye anions. In the analytical series with aminated sorbents, the Langmuir 2 model showed a better fit to the experimental data than the Langmuir 1 model. This finding indicates that at least 2 different sorption sites played an important role in the sorption of anionic dyes onto NEN1, NEN2, and NEN3. For NEN1, these active sites could be protonated hydroxyl groups (–OH_2_^+^) and protonated primary amino groups (–NH_3_^+^), and in the case of NEN2 and NEN3 also protonated secondary amino groups (–NH_2_^+^–).

The maximum sorption capacities determined for the sorbents tested are listed in Table 5. In the analytical series with RB5, the sorption capacities of newsprint after its pre-activation with epichlorohydrin and subsequent amination with ammonia (ammonia water), ethylenediamine, and diethylenetriamine increased by 2467%, 2747%, and 3151%, respectively, compared to the unmodified paper. In the case of RY84, the sorption capacity of NEN1, NEN2, and NEN3 was 941%, 956%, and 1202% higher compared to N, respectively. As mentioned in Section 2.3, the high efficiency of the aminated sorbents is due to the presence of a considerable amount of amine functional groups in the sorbent’s structure, which are important sorption sites for anionic dyes. The values of the K_1_ constants determined from the model indicate that the affinity of the dyes for the active sites of N and NE (–OH_2_^+^) was also generally lower than for the sorption centers of NEN1, NEN2, and NEN3 (–NH_3_^+^/–NH_2_^+^–).

The activation of newsprint paper with epichlorohydrin itself had no significant effect on its sorption capacity. The sorption capacity of NE towards RB5 and RY84 was 11% and 17% higher than that of N, respectively. Although NEN2 and NEN3 contain about 87% and 194% more amine groups than NEN1 (as shown by the analysis of nitrogen content, Table 1, Section 2.1), their sorption capacities do not differ that much. The sorption capacity of NEN2 towards RB5 and RY84 was only 10.9% and 1.5% higher than that of NEN1, while NEN3 was 26.7% and 25.1% more efficient in RB5 and RY84 sorption. The result obtained might be due to the fact that the additional amine groups in NEN2 and NEN3 (compared to NEN1) are mainly secondary amino groups (Figure 1). As mentioned in the Introduction section, sorption centers in the form of secondary amino groups may be characterized by poorer accessibility to sorbates than the primary amino groups. However, the size of the dye molecules could be of greater importance in this case. The molar mass of RB5 and RY84 is 991 g/mol and 1628 g/mol, respectively. Due to the small distances between the amine groups within an ethylamine or diethylenetriamine molecule (Figure 1), there is a high risk that the potential of many amine functional groups as sorption centers will not be utilized. For example, a dye molecule bound to a protonated primary amino group may restrict the access of other dye molecules to a nearby protonated secondary amino group due to its size. In summary, the amination of a sorbent material with ethylenediamine or diethylenetriamine can result in a larger number of amine functional groups being introduced into the sorbent than in the case of ammonia (ammonia water). However, a larger number of active sites does not necessarily mean a greater sorption capacity in a linear fashion (especially in the case of sorbates with a high molecular weight).

As in the studies on the kinetics of dye sorption, no clear correlations were found between the specific surface area of the sorbent materials tested, their porosity (Table 2), and their maximum sorption capacity for dyes. In the case of waste paper-based sorbents, the chemical composition of the sorbent material (the presence of ionizable functional groups) is probably of much greater importance for the sorption of anionic dyes.

Table 6 and Table 7 compare RB5 and RY84 sorption efficiencies on various lignocellulosic sorbents and activated carbons. The literature review shows that the amination of lignocellulosic sorbents, preceded by material activation with epichlorohydrin, significantly improves their sorption capacity for anionic dyes. In the case of the amination of materials without their pre-modification with epichlorohydrin, the increase in sorption capacity is relatively small (Table 6 and Table 7). As confirmed by data from the present study, sorbent modification with epichlorohydrin alone also does not significantly improve the sorption properties of the material.

Aminated sorbents based on newsprint paper (pre-activated with epichlorohydrin) have a much higher sorption capacity of RB5 and RY84 than the unmodified plant-based biosorbents. The aminated sorbents analyzed in this study show a higher sorption efficiency than the previously tested aminated sorbents of plant origin, such as sunflower and rapeseed hulls or wheat straw. The result obtained could be due to the very high content of polysaccharides (cellulose and hemicellulose) and lignin in the newsprint, i.e., components susceptible to reaction with epichlorohydrin, as well as to the specific structure of the material, characterized by good accessibility of its functional groups. As a result, a properly prepared aminated sorbent based on newsprint may have a significantly greater amount of amine functional groups on its surface than other biosorbents that have undergone the same chemical modifications, which translates into better dye sorption performance.

It is interesting to note that the aminated sorbents analyzed in this study (NEN2 and NEN3) exhibited higher sorption capacity than the commercial activated carbon (Table 6). This finding indicates their suitability as substitutes for commercial sorbents. The price of used (printed) newsprint paper, which is the basic material for producing these sorbents, is very low. Furthermore, the relatively small differences between the sorption capacities of the sorbents aminated with ammonia/ammonia water, ethylenediamine, or diethylenetriamine argue in favor of using the cheapest aminating agent in the production of sorbents.

Taking into account the approximate prices of materials used to produce NEN1 tested in this study (based on data from 2024 from the following websites: www.businessanalytiq.com, accessed on 3 December 2024; www.letsrecycle.com, accessed on 3 December 2024; www.paperindex.com, accessed on 3 December 2024), waste newsprint 50.00–250.00 USD/MT [93,94]; epichlorohydrin 1000–1400 USD/MT [95]; ammonia 350–590 USD/MT [96] and energy costs 0.05–0.40 USD/kWh (www.energybot.com, accessed on 3 December 2024; www.globalpetrolprices.com, accessed on 3 December 2024) [97,98], the estimated cost of producing 1 ton of NEN1 would be 600–1200 USD/MT (the calculation assumes that 0.25 to 0.4 tons of epichlorohydrin and ammonia are used per 1 ton of waste paper). Such a sorbent could therefore be cheaper than activated carbon, which costs USD 1500–2400/MT [99].

The issue of the disposal of the spent sorbent materials should be discussed as well. Theoretically, the regeneration of paper-based sorbents, which consists of the desorption of dyes, would be problematic and unprofitable. Such a process would have to be initiated with strong bases or other expensive chemical reagents and would also lead to the generation of a new batch of wastewater. In addition, paper materials have a limited shelf life and could be damaged or destroyed after several regeneration cycles.

One way to develop the spent paper-based sorbent could be to carbonize and activate it to produce full-grade activated carbon. However, this process would be quite energy-intensive, and the amount of the product obtained would be relatively small. Since paper and dyes have a relatively high calorific value, it seems more advantageous to dry and burn such a sorbent (e.g., in a heating plant) to recover energy from it. An alternative way to recover energy from the spent sorbents could also be their conditioning to increase their biodegradability, followed by their methane fermentation [100].

## 3. Materials

### 3.1. Waste Newsprint Paper

The newsprint paper waste came from outdated Polish newspapers. The newspapers were purchased from Polish press offices in Olsztyn (Poland) between 1 April and 30 April 2024. The total number of newspapers purchased was 78. The newspapers had a grammage of 50 g/m^2^. The approximate composition of the collected newsprint was as follows: cellulose 38.0–55.0%, hemicellulose 18.0–40.0%, lignin 18.0–30.0%, and other components (fillers, adhesives, and printing inks) 1.0–6.0% (based on literature data [101,102]).

### 3.2. Dyes

**Figure 8:**
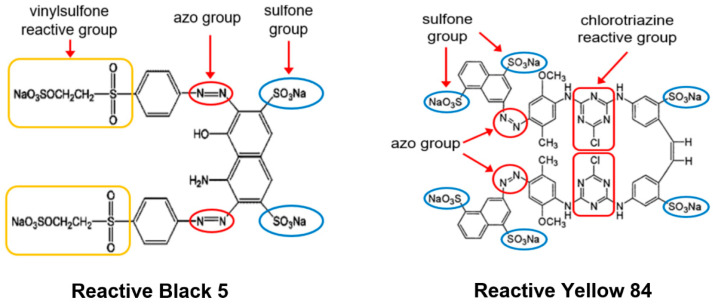
Chemical structure of Reactive Black 5 and Reactive Yellow 84 dyes.

The industrial dyes Reactive Black 5 (RB5) and Reactive Yellow 84 (RY84) (Figure 8) were purchased from the dye-producing plant “BORUTA-ZACHEM KOLOR SA” in Zgierz (Poland). The most important parameters of the dyes are listed in Table 8.

### 3.3. Chemical Reagents

The properties of the chemical reagents used in the study are listed in Table 9.

All chemical reagents used were purchased from POCH S.A., Gliwice, Poland, and were of p.a. (analytical purity) grade or higher.

### 3.4. Laboratory Equipment

The following laboratory equipment was used in the study:

- HI 110 pH meter (HANNA Instruments, Olsztyn, Poland)—for measuring and correcting the pH value of solutions;
- Water bath shaker type 357 (Elpin-Plus, Lubawa, Poland)—for modifying the sorbent with epichlorohydrin or aminating agents;
- Laboratory shaker SK-71 (JEIO TECH, Daejeon, Republic of Korea)—for dye sorption studies;
- Multi-Channel Stirrer MS-53M (JEIO TECH, Daejeon, Republic of Korea)—for dye sorption studies;
- UV-3100 PC spectrophotometer (VWR Spectrophotometer, VWR International LLC., Mississauga, ON, Canada)—for analyzing dye concentration in solutions;
- FT/IR-4700LE FT-IR spectrometer with single reflection ATR attachment (JASCO International, Tokyo, Japan)—for the generation of FTIR spectra of sorbents;
- FLASH 2000 Analyzer (Thermo Scientific, Waltham, MA, USA)—for elemental analysis and measurement of carbon and nitrogen contents.
- ASAP 2020 (Micromeritics, Norcross, GA, USA)—for measuring the porosity and surface area of sorbents.

## 4. Methods

### 4.1. Preparation of a Sorbent Based on Waste Newsprint Paper (N)

Newspapers were purchased for the sole purpose of conducting this study. The collected material did not contain any other contaminants apart from the standard composition of printed newsprint. For this reason, the newsprint was not pre-cleaned in any way prior to testing. All copies of the newspaper were shredded in an office shredder into 2 × 15 mm pieces, after which the shredded mass was mixed to standardize the batch of the material. The newsprint (N) based sorbent prepared this way was stored in tight polypropylene containers at a temperature of 25 °C.

### 4.2. Preparation of Newsprint Paper Modified with Epichlorohydrin (NE)

**Figure 9:**
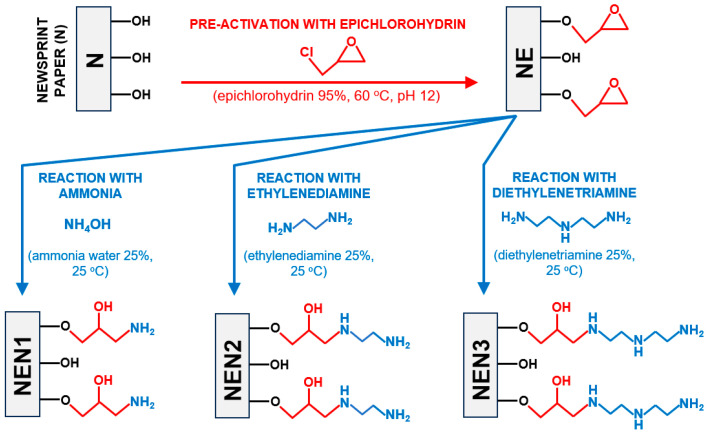
Scheme of the preparation of NE, NEN1, NEN2 and NEN3.

The shredded newsprint paper (N) (prepared according to the procedure described in Section 4.1) was weighed into an Erlenmeyer flask (capacity 500 mL) in an amount of 20.00 g dry matter (DM). A solution of epichlorohydrin (200 mL) with pH = 12 was added to the flask (the epichlorohydrin content in the prepared solution was not less than 95%). The flask was then secured with parafilm and placed on a shaker with a water bath set at 60 °C, a stirring speed of 100 rpm, and an oscillation amplitude of 30 mm for 12 h. After the specified time, the resulting paper pulp was separated from the solution on a laboratory sieve and then rinsed with a large volume of deionized water to remove unreacted epichlorohydrin from the material. The paper pulp was then dried in a laboratory dryer (105 °C). The resulting epichlorohydrin (NE) modified/activated newsprint pulp was stored in a sealed container at 25 °C. A simplified scheme of NE preparation is shown in Figure 9.

### 4.3. Preparation of Aminated Newsprint Paper (NEN1, NEN2, NEN3)

The paper modified/activated with epichlorohydrin (NE), obtained according to the procedure described in Section 4.2, was weighed in the amount of 20.00 g DM into a conical flask (capacity 500 mL). Then, 200 mL of a 25% aminating agent solution (ammonia, ethylenediamine, or diethylenetriamine) was added to the vessel. The cork was protected with parafilm, and the vessel was placed on a shaker (100 rpm, vibration amplitude 30 mm). After 12 h of amination, the paper was separated from the solution and washed on a laboratory sieve with a large volume of deionized water to remove the unreacted aminating agent. After washing, the modified paper was dried in a laboratory dryer (105 °C). The materials obtained in this way (NEN1, NEN2, and NEN3) were stored in a sealed container at a temperature of 25 °C, similar to N and NE. A simplified scheme of the preparation of the aminated sorbents is presented in Figure 9.

This work does not present data from analyses of the sorption properties of newsprint modified directly with aminated agents (without pre-activation of the sorbent surface with epichlorohydrin). This is due to the very low efficiency of the direct amination of lignocellulosic materials demonstrated in previous studies [53,54,55,56].

### 4.4. Studies on the Influence of pH on the Efficiency of Dye Sorption

The sorbent (2.50 g DM) was weighed on a precision balance into a series of laboratory beakers (600 mL capacity). The previously prepared dye solutions (500 mL) with a concentration of 100 mg/L and pH values between 2 and 11 were then added to the beakers. The beakers were placed on a multistage magnetic stirrer for 2 h. Afterwards, samples of the solutions (10 mL) were taken from the beakers into the previously prepared polypropylene test tubes using an automatic pipette. The pH value of the solutions was also measured at the end of the experiment.

### 4.5. Studies on the Kinetics of Dye Sorption

The sorbent (5.00 g DM) was weighed into laboratory beakers (1000 mL capacity). The previously prepared dye solutions (1000 mL) with a concentration of 1000 mg/L and optimal sorption pH (determined based on the studies described in Section 4.4) were added to the beakers. The beakers were then placed on a multistage magnetic stirrer. At specific time intervals (i.e., after 0, 10, 20, 30, 45, 60, 90, 120, 150, 180, 210, and 240 min), samples of the solutions (2 mL) were collected from the beakers into the previously prepared test tubes using automatic pipettes.

### 4.6. Studies on the Maximum Sorption Capacity of the Sorbents

The sorbents (2.50 g DM) were weighed into a series of beakers (600 mL capacity). Sorbents and then dye solutions (500 mL) were added to the beakers at concentrations ranging from 10 mg/L to 1000 mg/L (for the series of experiments with RB5 and NEN1, NEN2 and NEN3, the range of concentrations applied was 10–1600 mg/L) and optimum pH (determined based on the studies described in Section 4.4). The beakers were placed on a multistage magnetic stirrer for the reaction equilibrium time (determined based on the studies described in Section 4.5). Once the sorption had been completed, samples of the solutions (10 mL) were collected from the flasks and transferred to the prepared test tubes.

Notes to Section 4.4, Section 4.5 and Section 4.6

- The sorption properties of each of the 5 sorbents tested (N, NE, NEN1, NEN2, and NEN3) were analyzed using 2 dyes (RB5 and RY84).
- All dye solutions were prepared with deionized water.
- pH values of the solutions were corrected by adding small amounts of 1 M HCl or 1 M NaOH under continuous pH measurement.
- The portions of sorbents added to the flasks or beakers were prepared on a precision balance with an accuracy of 0.001 g.
- The sorbent dose was 5.00 g/L in all test series.
- The selected mixing parameters on a multistage magnetic stirrer (200 rpm, magnetic stirrer with a Teflon coating of size 50 × 8 mm) ensured a uniform distribution of the sorbent throughout the entire volume of the solution during the process.
- The concentrations of the dyes remaining in the solution were determined spectrophotometrically using a UV-VIS spectrophotometer with a cuvette with an optical path length of 10 mm.
- The calibration curves plotted for the RB5 and RY84 dyes, entered into the spectrophotometer, enabled determinations of solutions with concentrations of 0–50 mg/L. Solutions with higher concentrations were diluted with deionized water.
- For each sorbent tested, an additional series of experiments were carried out without the dye. The aqueous solutions obtained in this way were used to calibrate the spectrophotometer before the dye concentration was analyzed. In this way, measurement errors due to possible color impurities released into the solutions by the sorbents tested could be eliminated.
- All analytical series were carried out in triplicate.
- A constant temperature of 25 °C was maintained in the laboratory during analyses.

### 4.7. FTIR Analysis of the Sorbents

The FTIR spectra of the sorbents tested were achieved using an FT/IR-4700LE spectrometer with a single reflecting ATR diamond crystal (JASCO International, Tokyo, Japan). The material samples were scanned in the wavelength range from 4000 cm^−1^ to 400 cm^−1^. The resolution of the spectra was 1 cm^−1^. The spectrum of each sample was generated based on the average values of 64 measurements. Before each measurement, the ATR diamond crystal was carefully cleaned with acetone and dried with a paper towel, and the baseline was corrected.

### 4.8. Measurement of the Specific Surface Area and Porosity of the Sorbents

The specific surface area and porosity of the sorbents tested were measured using the ASAP 2020 instrument (Micromeritics, Norcross, GA, USA). The analyses were performed using the low-temperature nitrogen adsorption/desorption method. Prior to analysis, samples were degassed under a vacuum at 100 °C for 4 h. The measurement results were reported with an accuracy of three significant digits.

### 4.9. Calculation Methods

The amount of dye adsorbed on the tested sorbents was determined from Formula (1):

(1) $$Q_{S}=(C_{0}-C_{S})\times \frac{V}{m}$$

Q_S_—mass of sorbed dye [mg/g];

C_0_—initial concentration of dye [mg/L];

C_S_—concentration of dye after sorption [mg/L];

V—volume of the solution [L];

m—mass of the sorbent [g].

The sorption kinetics of the dyes on the sorbents tested were described with pseudo-first-order (2), pseudo-second-order (3), and intraparticle diffusion models (4).

(2) $$q=q_{e}\times (1-e^{\left(-k_{1}\times t\right)})$$

(3) $$q=\frac{\left(k_{2}\times {q_{e}}^{2}\times t\right)}{\left(1+k_{2}\times q_{e}\times t\right)}$$

(4) $$q=k_{id}\times t^{0.5}$$

q—instantaneous value of sorbed dye [mg/g];

q_e_—the amount of dye sorbed at the equilibrium state [mg/g];

t—time of sorption [min];

k_1_—pseudo-first-order adsorption rate constant [1/min];

k_2_—pseudo-second-order adsorption rate constant [g/(mg×min)];

k_id_—intraparticle diffusion model adsorption rate constant [mg/(g×min^0.5^)].

Experimental data from maximum sorption capacity studies (N, NE, NEN1, NEN2, and NEN3) with respect to RB5 and RY84 were described using the three most popular sorption models: Langmuir isotherm 1 (5), Langmuir isotherm 2 (Langmuir double isotherm) (6), and Freundlich isotherm (7).

(5) $$Q=\frac{\left(Q_{max}\times K_{C}\times C\right)}{\left(1+K_{C}\times C\right)}$$

(6) $$Q=\frac{\left(b_{1}\times K_{1}\times C\right)}{\left(1+K_{1}\times C\right)}+\frac{\left(b_{2}\times K_{2}\times C\right)}{\left(1+K_{2}\times C\right)}$$

(7) $$Q=K\times C^{\frac{1}{n}}$$

Q—mass of sorbed dye [mg/g];

Q_max_—maximum sorption capacity in Langmuir equation [mg/g];

b_1_—maximum sorption capacity of sorbent (type I active sites) [mg/g];

b_2_—maximum sorption capacity of sorbent (type II active sites) [mg/g];

K_C_—constant in Langmuir equation [L/mg];

K_1_, K_2_—constants in Langmuir 2 equation [L/mg];

K—the equilibrium sorption constant in the Freundlich model;

n—Freundlich equilibrium constant;

C—concentration of the dye remaining in the solution [mg/L].

## 5. Conclusions

The efficiency of newsprint amination depends largely on the type of an aminating agent. By analyzing the nitrogen content in the modified materials, it was found that when the sorbent was aminated with ethylenediamine and diethylenetriamine, 87% and 194% more amine groups were introduced into the material than upon its amination with ammonia (ammonia water).

The amination of newsprint paper, which is preceded by material pre-activation with epichlorohydrin, considerably improves its sorption properties towards anionic dyes. In the case of the RB5 dye, the sorption capacity of the tested materials after pre-activation and amination with ammonia, ethylenediamine, and diethylenetriamine increased by 2467%, 2747%, and 3151%, respectively.

The sorption capacities achieved by the aminated sorbents are not proportional to the number of functional groups introduced during amination. In the case of sorbents modified with ethylenediamine or diethylenetriamine, the unused potential of many amine functional groups as sorption centers may result from the small distances between these groups as well as a relatively large molar mass of the dyes, which limit access to them due to their large size.

The activation of newsprint with epichlorohydrin leads to the introduction of epoxy groups into the material. However, the effect of this single modification on the sorption capacity of the paper is small. The sorption capacity of the paper activated with epichlorohydrin towards RB5 and RY84 was only 11% and 17% higher than that of the unmodified material.

The pH of the solutions has a significant influence on the sorption efficiency of anionic dyes. The sorption of RB5 and RY84 on the tested sorbents was the highest at low pH (pH 2–3). In general, the sorption efficiency of the dyes decreased with increasing pH of the solutions.

The equilibrium time of RB5 and RY84 sorption depends on the sorbent type. The sorption of these dyes on the unmodified newsprint and the paper modified with epichlorohydrin took between 120 and 150 min, while their sorption on the aminated materials took 90 min. The shorter sorption times obtained for the aminated sorbents are probably due to the greater number of protonated functional groups. The strong overall positive charge on the surface of the aminated materials significantly accelerated the process of anionic dye binding.

The sorption of RB5 and RY84 onto the newsprint-based sorbents occurs in two main phases, which differ in intensity and duration.

Only one type of sorption center played the main role in the sorption of anionic dyes on the unmodified newsprint and on the paper modified with epichlorohydrin. Presumably, this type of active site was the protonated hydroxyl groups that interacted electrostatically with the dye anions. In the case of the aminated paper-based sorbents, at least two different types of sorption centers played the main role in RB5/RY84 sorption; these could probably be protonated hydroxyl groups and protonated primary and secondary amino groups.

The disadvantage of the aminated sorbents tested in this study is the necessity of using expensive epichlorohydrin (activating agent) for their preparation. It will therefore be advisable to develop a more economical method for the amination of materials in the future.

## Figures and Tables

**Table 1:** Nitrogen, carbon, and hydrogen contents in the tested sorbents. Analyses were carried out on an elemental analyzer—FLASH 2000 (THERMO SCIENTIFIC, USA) (three repetitions of measurements).

Tested Sorbent	Nitrogen Content [%]	Carbon Content [%]	Hydrogen Content [%]	Approximate Amount of Nitrogen Introduced During Amination [%] (Based on Average Values)
N	0.64 ± 0.07	46.08 ± 0.16	6.14 ± 0.10	-
NE	0.68 ± 0.12	45.12 ± 0.18	6.10 ± 0.09	-
NEN1	1.34 ± 0.03	45.88 ± 0.28	6.34 ± 0.09	0.70
NEN2	1.94 ± 0.09	45.41 ± 0.17	6.39 ± 0.05	1.30
NEN3	2.70 ± 0.18	46.01 ± 0.10	6.36 ± 0.10	2.03

**Table 2:** Results of measurements of low-temperature nitrogen adsorption/desorption (BET).

Tested Sorbent	BET Surface Area [m^2^/g]	Pore Volume [cm^3^/g]	Pore Size (Average) [nm]
N	0.488 ± 0.0331	0.00212	25.0
NE	0.389 ± 0.0331	0.00495	37.9
NEN1	0.499 ± 0.0331	0.00327	20.8
NEN2	0.528 ± 0.0472	0.00311	24.6
NEN3	0.377 ± 0.0350	0.00378	23.7

**Table 3:** Kinetic parameters of RB5 and RY84 sorption onto N, NE, NEN1, NEN2, and NEN3, determined from the pseudo-first-order and pseudo-second-order models (based on the average of three measurements + sorption equilibrium time).

Tested Sorbent	Dye	Dye Conc.	Pseudo-First-Order Model			Pseudo-Second-Order Model			Exp. Data	Equil. Time
			k_1_	q_e,cal_.	R^2^	k_2_	q_e,cal_.	R^2^	q_e,exp_.	
		[mg/L]	[1/min]	[mg/g]	-	[g/mg × min]	[mg/g]	-	[mg/g]	[min]
N	RB5	1000	0.0610	6.06	0.9954	0.0134	6.70	0.9971	6.22	120
	RY84	1000	0.0357	9.06	0.9784	0.0043	10.42	0.9943	9.42	150
NE	RB5	1000	0.0485	6.62	0.9915	0.0087	7.49	0.9978	6.76	120
	RY84	1000	0.0368	11.49	0.9879	0.0036	13.17	0.9971	11.86	150
NEN1	RB5	1000	0.0532	162.24	0.9802	0.0004	181.50	0.9957	161.25	90
	RY84	1000	0.0447	99.60	0.9915	0.0005	113.56	0.9961	100.41	90
NEN2	RB5	1000	0.0460	174.40	0.9854	0.0003	197.73	0.9960	176.17	90
	RY84	1000	0.0511	101.51	0.9757	0.0006	113.72	0.9936	102.63	90
NEN3	RB5	1000	0.0489	192.22	0.9841	0.0003	216.51	0.9949	195.31	90
	RY84	1000	0.0501	105.35	0.9862	0.0006	118.56	0.9960	106.36	90

**Table 4:** Rate constants of RB5 and RY84 diffusion determined from a simplified intraparticle diffusion model.

Tested Sorbent	Dye	Dye Conc.	Phase 1			Phase 2		
			k_d1_	Duration	R^2^	k_d2_	Duration	R^2^
		[mg/L]	[mg/(g × min^0.5^)]	[min]	-	[mg/(g × min^0.5^)]	[min]	-
N	RB5	1000	0.903	30	0.9981	0.145	90	0.9817
	RY84	1000	1.117	30	0.9978	0.482	120	0.9907
NE	RB5	1000	0.899	30	0.9974	0.242	90	0.9961
	RY84	1000	1.403	30	0.9998	0.534	120	0.9777
NEN1	RB5	1000	24.587	20	0.9996	11.050	70	0.9964
	RY84	1000	13.733	20	0.9964	7.192	70	0.9891
NEN2	RB5	1000	24.871	20	0.9999	13.259	70	0.9933
	RY84	1000	15.254	20	0.9965	7.264	70	0.9853
NEN3	RB5	1000	28.075	20	0.9999	14.258	70	0.9898
	RY84	1000	15.547	20	0.9999	7.380	70	0.9845

**Table 5:** Constants determined from Langmuir 1, Langmuir 2, and Freundlich models.

Tested SorBent	Dye	Langmuir 1 Model			Langmuir 2 Model						Freundlich Model		
		Q_max_	K_c_	R^2^	Q_max_	b_1_	K_1_	b_2_	K_2_	R^2^	k	n	R^2^
		[mg/g]	[L/mg]	-	mg/g	mg/g	L/mg	mg/g	L/mg	-	-	-	-
N	RB5	7.12	0.0060	0.9913	7.12	3.57	0.0060	3.55	0.0060	0.9913	0.458	0.388	0.9422
	RY84	10.24	0.0190	0.9931	10.24	5.77	0.0190	4.47	0.0190	0.9931	1.967	0.247	0.8753
NE	RB5	7.89	0.0057	0.9960	7.89	4.02	0.0057	3.87	0.0057	0.9960	0.484	0.394	0.9498
	RY84	11.95	0.0201	0.9945	11.94	5.93	0.0201	6.01	0.0201	0.9945	2.323	0.247	0.8810
NEN1	RB5	176.46	0.0905	0.9841	182.78	147.05	0.0477	35.73	1.3001	0.9967	44.462	0.233	0.9107
	RY84	101.64	0.0603	0.9955	106.58	95.77	0.0664	10.81	0.0360	0.9960	21.067	0.272	0.9089
NEN2	RB5	188.23	0.2355	0.9930	202.70	169.85	0.0550	32.85	0.2932	0.9961	58.159	0.207	0.8589
	RY84	105.71	0.0720	0.9966	108.15	99.84	0.0783	8.31	0.0075	0.9968	23.333	0.265	0.8988
NEN3	RB5	227.08	0.2334	0.9946	231.50	186.48	0.1460	45.02	1.3704	0.9984	67.530	0.226	0.8715
	RY84	105.94	0.1076	0.9929	133.31	100.79	0.1179	32.52	0.0007	0.9940	26.953	0.244	0.8963

**Table 6:** Comparison of the sorption properties of various lignocellulosic sorbents and activated carbons towards RB5 (literature data).

Sorbent	Q_max_ [mg/g]	pH of Sorption	Time of Sorption [min]	Source
Newsprint paper aminated with diethylenetriamine (pre-activated with epichlorohydrin) (NEN3)	231.5	3	90	This work
Newsprint paper aminated with ethylenediamine (pre-activated with epichlorohydrin) (NEN2)	202.7	3	90	This work
Activated carbon Filtrasorb 400 (commercial)	198.0	5.2	720	[79]
Newsprint paper aminated with ammonia (pre-activated with epichlorohydrin) (NEN1)	182.8	3	90	This work
Rapeseed hulls—aminated with ammonia (pre-activated with epichlorohydrin)	135.8	3	120	[55]
Activated carbon (powder)	125.8	2	240	[71]
Wheat straw aminated with ammonia (pre-activated with epichlorohydrin)	91.0	3	210	[56]
Buckwheat hulls aminated with ammonia (pre-activated with epichlorohydrin)	85.2	3	300	[53]
Goldenrot biomass aminated with ammonia (pre-activated with epichlorohydrin)	71.3	3	120	[54]
Activated carbon modified with SPC	69.9	2	<60	[80]
Activated carbon—powdered	58.8	-	-	[81]
Sunflower seed shells aminated with ammonia (pre-activated with epichlorohydrin)	51.0	3	240	[26]
Activated carbon from bamboo	39.0	2	60	[77]
Activated carbon from Carob tree	36.9	2	120	[76]
Biochar from gasification residues	35.7	-	90	[78]
Rape stalks (waste)	32.8	2.5	30	[82]
Banana peel (powder)	26.9	3	60	[83]
Rapeseed hulls aminated with ammonia (without activation)	26.3	3	150	[55]
Activated carbon from palm shell	25.1	2	300	[84]
Wood (walnut) activated carbon	19.3	5	400	[85]
Wheat straw aminated with ammonia (without activation)	17.5	3	210	[56]
Wheat straw	15.7	7	195	[56]
Rapeseed husks	15.2	3	180	[55]
Beech sawdust	13.9	3	1440	[74]
Seed scales of *Eriobotrya japonica*	13.8	3	150	[86]
Cotton seed husks	12.9	2	30	[87]
Goldenrot biomass aminated with ammonia (without activation)	10.6	3	150	[54]
Newsprint paper activated with epichlorohydrin (NE)	7.9	3	120	This work
Buckwheat hulls aminated with ammonia (without activation)	7.4	3	300	[53]
Newsprint paper (N)	7.1	3	120	This work
Buckwheat hulls	4.4	3	300	[53]
Sunflower seed hulls	2.9	3	210	[26]
Cotton fibers	2.7	3	240	[52]
Goldenrot biomass	2.3	3	150	[54]
Macadamia seed husks	1.2	3	510	[88]
Sunflower biomass	1.1	2	210	[89]
Pumpkin seed husks	1.0	3	60	[28]
Coconut shells	0.8	2	60	[90]

**Table 7:** Comparison of the sorption properties of various lignocellulosic sorbents and activated carbons towards RY84 (literature data).

Sorbent	Q_max_ [mg/g]	pH of Sorption	Time of Sorption [min]	Source
Newsprint paper aminated with diethylenetriamine (pre-activated with epichlorohydrin) (NEN3)	133.3	3	90	This work
Rapeseed husks aminated with ammonia (activated with epichlorohydrin)	114.2	3	120	[55]
Newsprint paper aminated with ethylenediamine (pre-activated with epichlorohydrin) (NEN2)	108.2	3	90	This work
Newsprint paper aminated with ammonia (pre-activated with epichlorohydrin) (NEN1)	106.6	3	90	This work
Sunflower seed hulls aminated with ammonia (pre-activated with epichlorohydrin)	63.3	3	240	[26]
Goldenrot biomass aminated with ammonia (pre-activated with epichlorohydrin)	59.3	3	120	[54]
Cotton fibers aminated with ammonia (pre-activated with epichlorohydrin)	43.3	2	240	[52]
Activated carbon from the Borassus flabellifer plant	40.0	-	-	[91]
Rapeseed husks aminated with ammonia (without activation)	19.7	3	150	[55]
Cotton fibers	15.9	2	240	[52]
Rapeseed husks	13.7	3	180	[55]
Newsprint paper activated with epichlorohydrin (NE)	11.9	3	150	This work
Wool	11.0	7	180	[92]
Newsprint paper (N)	10.2	3	150	This work
Sunflower seed hulls aminated with ammonia (without activation)	9.9	3	180	[26]
Sunflower seed hulls	4.2	3	90	[26]
Goldenrot biomass	2.3	3	180	[54]
Compost	2.2	3	180	[72]

**Table 8:** Characteristics of dyes used in the research.

Dymolecules-29-06024e Name	Reactive Black 5 (RB5)	Reactive Yellow 84 (RY84)
Chemical formula	C_26_H_21_N_5_Na_4_O_19_S_6_	C_56_H_38_Cl_2_N_14_Na_6_O_20_S_6_
Molecular weight	991.0 g/mol	1628.0 g/mol
Dye type	anionic (reactive)	anionic (reactive)
Dye class	double azo dye	double azo dye
Type of reactive groups	vinylsulfone	chlorotriazine
λ_max_	600 nm	356 nm
Uses	dyeing of cotton, viscose, wool	dyeing of polyester, cotton, synthetic silk
Other trade names	Begazol Black B; Remazol Black B	Active Yellow HE-4R; Lamafix Yellow HER.
Purity of the commercial product	dye content 70%	dye content 97%

**Table 9:** Characteristics of chemical reagents used in the study.

Name	Structural Formula (Simplified)	Molecular Weight [g/mol]	Purity	Application in This Study
Hydrochloric acid	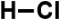	36.5	37%	diluted, for the correction of the pH of solutions
Sodium hydroxide (microgranules)	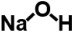	40.0	99.9%	in the solution form, for the correction of the pH of solutions
Epichlorohydrin	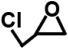	92.5	>99%	for the modification/activation of sorbent
Ammonia (ammonia water)	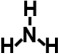	17.0	25.0% (water solution)	for the amination of sorbent
Ethylenediamine	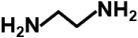	60.1	>99%	for the amination of sorbent
Diethylenetriamine	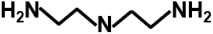	103.2	>99%	for the amination of sorbent

## Data Availability

The data presented in this study are available upon request from the corresponding author.
